# Danggui Niantong granules in the treatment of rheumatoid arthritis - a mechanistic approach using ceRNA networks

**DOI:** 10.3389/fphar.2026.1718584

**Published:** 2026-02-06

**Authors:** Yisi Cai, Yanyan Song, Xiaoling Zeng, Gang Liu, Lixia Yuan

**Affiliations:** 1 School of Traditional Chinese Medicine, Southern Medical University, Guangzhou, China; 2 Third Level Research Laboratory of State Administration of Traditional Chinese Medicine, Guangdong Provincial Key Laboratory of Chinese Medicine Pharmaceutics, School of Traditional Chinese Medicine, Southern Medical University, Guangzhou, China; 3 Syndrome Laboratory of Integrated Chinese and Western Medicine, School of Traditional Chinese Medicine, Southern Medical University, Guangzhou, China; 4 Department of Rehabilitation Medicine, Nanfang Hospital, Southern Medical University, Guangzhou, China

**Keywords:** fibroblast-like synoviocytes, invasion, MH7A, migration, traditional Chinese medicine

## Abstract

**Context:**

Danggui Niantong granules (DGNTG), a traditional Chinese medicine, serve as an effective therapeutic agent for the treatment of rheumatoid arthritis (RA). However, the comprehensive molecular mechanisms of DGNTG in RA remain unclear. Increasing evidence highlights the significant role of competing endogenous RNAs (ceRNA) in diagnosing and treating various diseases.

**Objective:**

The study aims to explore the molecular mechanism of DGNTG in the treatment of RA through the ceRNA network.

**Materials and methods:**

The MH7A cells were divided into control group and DGNTG group (2 and 4 mg/mL).The proliferation, migration and invasion ability of MH7A were accessed using MTT assay, cloning formation, wound-healing assay transwell assay and Western blotting. Subsequently, ceRNA microarray analyses were performed and a circRNA-miRNA-mRNA ternary transcription network was established. The data were validated through qPCR.

**Results:**

DGNTG inhibited proliferation, suppressed migration and invasion in MH7A (*P < 0.05, 0.01 or  0.001*), with the most pronounced effects observed in the DGNTG (4 mg/mL) group. Subsequently, we identified 301 differentially expressed mRNAs and 507 differentially expressed circRNAs (FC ≤ 0.5 or ≥2, *P < 0.05*). Bioinformatics analyses indicated that DGNTG may exert therapeutic effects through multiple pathways. Furthermore, we constructed a circRNA-miRNA-mRNA network and conducted additional bioinformatics analysis on this network. In addition, we developed a representative ceRNA network and analyzed the correlations among its components.

**Discussion and conclusions:**

This study presents evidence that DGNTG exerts anti-RA effects through the inhibition of synoviocyte proliferation, migration and invasion. Additionally, It provided a valuable resource for elucidating the mechanism of action of DGNTG by constructing a competing endogenous RNA network based on transcriptomic data obtained from DGNTG-treated MH7A cells.

## 1 Introduction

Rheumatoid arthritis (RA), a systemic autoimmune disease, is characterized by the inflammatory proliferation of synovial tissue, infiltration of inflammatory cells, and progressive destruction of articular cartilage. In the early stages, patients typically experience joint swelling, stiffness and pain. As the disease advances, joint deformities may develop, leading to consequential loss of function. This sequela not only disrupts the patient’s daily life but also imposes a significant financial burden on families and society. Alarmingly, reports indicate that the prevalence of RA may be on the rise (Finckh et al., 2022). Currently, disease-modifying antirheumatic drugs (DMARDs), including nonsteroidal anti-inflammatory drugs, glucocorticoids and opioids, represent the primary treatments for RA. However, the therapeutic effectiveness of DMARDs is limited and often accompanied by considerable adverse effects such as hepatotoxicity (Ezhilarasan, 2021). Additionally, the risk of serious infections associated with new biological agents is elevated compared to conventional synthetic DMARDs, despite the ongoing emergence of these agents (Kerschbaumer et al., 2023; Sepriano et al., 2023). RA remains a challenging disease due to its complex pathogenesis (Radu and Bungau, 2021). Highlighting the urgent need for novel therapeutic approaches and targets. The use of Traditional Chinese Medicine (TCM) in treating RA is notable for its minimal adverse reactions and multi-target effects, thus attracting increased attention for its potential role in disease management.

Danggui Niantong Granules (DGNTG) is a medicinal preparation derived from Danggui Niantong Decoction (DGNTD), a classic Chinese medicine prescription for treating RA. This formula comprises 15 distinct Chinese herbs (Table 1). Utilizing evidence-based medicine, DGNTD demonstrates clear clinical efficacy and a reduced incidence of adverse reactions (Hu et al., 2020). Our previous research indicated that DGNTD can ameliorate synovial inflammation in adjuvant arthritis rats by inhibiting the infiltration of inflammatory cells, cartilage and bone destruction (Lu et al., 2022a). However, prior studies have primarily concentrated on individual targets, resulting in a limited understanding of the roles of functional RNA molecules and RNA-mediated regulatory networks in the mechanism of DGNTG in RA.

**TABLE 1:** The components of Danggui Niantong granules.

Chinese name	Scientific name	Authority	Family
Yin chen	*Artemisia capillaris*	Thunb	Asteraceae
Qiang huo	*Notopterygium incisum*	Ting ex H. T. Chang	Apiaceae
Zhu ling	*Polyporus umbellatus*	(Pers.) fr	Polyporaceae
Dang gui	*Angelica sinensis*	(Oliv.) Diels	Apiaceae
Ku shen	*Sophora flavescens*	Aiton	Fabaceae
Zhi mu	*Anemarrhena asphodeloides*	Bunge	Asparagaceae
Ge gen	*Pueraria lobata*	(Willd.) Ohwi	Fabaceae
Sheng ma	*Cimicifuga foetida*	L	Ranunculaceae
Gan cao	*Glycyrrhiza uralensis*	Fisch	Fabaceae
Ren shen	*Panax ginseng*	C.A. Mey	Araliaceae
Bai zhu	*Atractylodes macrocephala*	Koidz	Asteraceae
Fang feng	*Saposhnikovia divaricata*	(Turcz.) Schischk	Apiaceae
Cang zhu	*Atractylodes lancea*	(Thunb.) DC.	Asteraceae
Huang qin	*Scutellaria baicalensis*	Georgi	Lamiaceae
Ze xie	*Alisma plantago-aquatica*	L	Alismataceae

Competing endogenous RNAs (ceRNAs) is a novel class of molecular mechanisms that regulate gene expression through the competitive binding of microRNAs (miRNAs) (Salmena et al., 2011). The ceRNA hypothesis asserts that any RNA molecule can act as a competing endogenous RNA if it contains identical miRNA-responsive elements (MREs). This category encompasses long non-coding RNAs (lncRNAs), circular RNAs (circRNAs), pseudogenes, and messenger RNAs (mRNAs). Among these, circRNAs exhibit high stability, strong conservation, and abundant expression across various tissues. As a result, circRNAs are considered promising diagnostic and therapeutic targets for a range of diseases (Liu et al., 2022). In RA, circRNAs have been demonstrated to play a crucial role in early diagnosis and effective treatment (Jiang et al., 2023). The study reported that circCDKN2B−AS_006 enhances the proliferation, migration, and invasion of RA fibroblast-like synoviocytes by modulating the miR−1258/RUNX1 axis (Xu et al., 2023). Additionally, circRNA_09505 regulates macrophage inflammation by functioning as a ceRNA for miR-6089 via the AKT1/NF-κB signaling pathway (Yang et al., 2020). The circRNA-miRNA-mRNA transcriptional network, which includes multiple ceRNA axes, has emerged as a focal point for researchers investigating the pathophysiological mechanisms underlying RA.

Fibroblast-like synoviocytes (FLS) are among the primary pathological cell types implicated in the progression of RA. The hyperplasia and invasion of synovial tissues can lead to the formation of pannus, which subsequently destroys cartilage and bone (Nygaard and Firestein, 2020). Consequently, inhibiting the proliferation, migration, and invasion of synovial tissue represents a critical therapeutic target for managing RA. Our previous research demonstrated that DGNTG can alleviate the proliferation, migration and invasion of synovial tissue *in vivo* by inhibiting autophagy and promoting apoptosis (Lu et al., 2022b). However, the associated genes and pathways have not been explored in depth. Building on our previous finding, this study aims to conduct a comprehensive analysis of the relevant gene targets using high-throughput sequencing technology. The MH7A cell line, characterized by rapid proliferation and the ability to undergo multiple passages, is frequently employed in RA research. Therefore, we utilized the MH7A cell line for this investigation.

**FIGURE 1:**
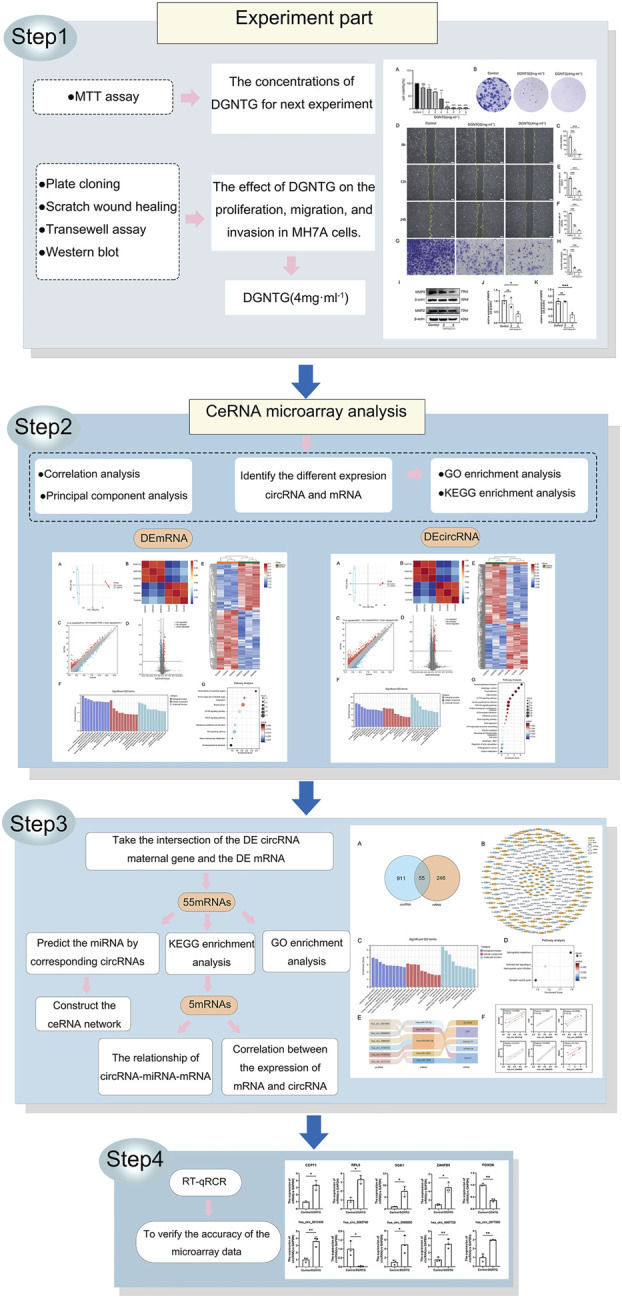
Workflow diagram of the study.

This study seeks to investigate the effects of DGNTG on RA and the circRNA-miRNA-mRNA transcriptional network in MH7A, with the goal of elucidating its molecular mechanisms in RA treatment. The workflow diagram is presented in Figure 1.

## 2 Materials and methods

### 2.1 Materials

Dulbecco’s Modified Eagle Medium (DMEM) was obtained from Wuhan Pricella Biotechnology Co., Ltd. (Wuhan, China). Fetal Bovine Serum (FBS) was sourced from ExCell Bio (Suzhou, China). Matrigel® Basement Membrane Matrix was acquired from Becton, Dickinson and Company (NV, USA). 24-well transwell inserts were procured from Corning (NY, USA). The Bradford protein assay kit and ColorMixed Protein Marker (Cat. FD0671) were purchased from Fdbio Science (Hangzhou, China). Beta Actin Recombinant antibody and Monoclonal rabbit antibodies MMP2 were obtained from Proteintech Group, Inc. (Wuhan, China), while the monoclonal rabbit antibody against MMP9 was sourced from Zenbio Science (Chengdu, China). The Goat Anti-Rabbit IgG (H + L) HRP and Goat Anti-Mouse IgG (H + L) HRP were obtained from Affinity Bioscirences (Jiangsu, China). Crystal Violet Staining Solution was acquired from Beyotime Biotechnology (Shanghai, China). ChamQ SYBR qPCR Master Mix, HiScript™ III RT SuperMix for qPCR, and Trizol were obtained from Nanjing Vazyme Biotech Co. Ltd. (Nanjing, China). All other chemicals and reagents were of analytical grade and procured from commercial sources.

### 2.2 Preparation of DGNTG

DGNTG was manufactured by Jiangxi Xinglin Baima Pharmaceutical Co., Ltd. (Jiangxi, China) including 15 kinds of Chinese herbs (Table 1). Our research group employed a high-performance liquid chromatography system for the quality control of DGNTG, revealing that baicalin was the primary component of DGNTG (Lu et al., 2022c). To ensure complete dissolution, DGNTG was added to DMEM medium at a concentration of 20 mg/mL, and the solution was used immediately.

### 2.3 Cell culture

The human rheumatoid arthritis synovial cell line MH7A (*Homo sapiens*, CVCL_0427) was obtained from FuHeng BioLogy (Shanghai, China) (Cat. CBP61649). MH7A cells were cultured in DMEM supplemented with 10% FBS and 1% penicillin-streptomycin, and maintained in a controlled-temperature incubator at 37 °C with 5% CO2.

### 2.4 MTT assay

The Methylthiazolyldiphenyl-tetrazolium bromide (MTT) assay was utilized to evaluate cell proliferation. Cells were seeded in a 96-well plate at a density of 5 × 10^3^ cells/well. After 24 h, the cells were treated with DGNTG at various concentrations, ranging from 0 to 8 mg/mL, for an additional 24 h. The MTT solution was prepared by mixing it with DMEM in a 1:10 ratio, and 100 μL of this mixture was added to each well. Following a 4 h incubation, the culture medium in each well was removed, and 150 μL of DMSO was added to each well and shaken for 20 min to ensure complete dissolution of the crystals before measuring absorbance at 490 nm using a microplate reader (Thermo Scientific FC, USA). Relative cell viability was expressed as a percentage of the control group.

### 2.5 Colony formation assay

MH7A cells were plated into a 6-well cell culture plate at a density of 500 cells per well and cultured in DMEM supplemented with 10% FBS with or without DGNTG (2 and 4 mg/mL) for a duration of 2 weeks. After incubation, the MH7A cells were fixed in methanol for 20 min and subsequently stained with 0.1% crystal violet for 30 min at room temperature. The number of colonies (>50 cells) was counted using an ImmunoSpot S5 Versa Analyzer (Cell Technology Ltd., USA).

### 2.6 Wound healing assay

For the wound healing assay, MH7A cells were seeded at a density of 8 × 10^5^ cells per well in 6-well plates. Once the cells reached a confluent monolayer, a sterile pipette tip was employed to create a vertical scratch at the center of the well, thereby generating the wound. Following a wash with PBS, the cells were cultured in a basic medium (DMEM) supplemented with 2% FBS. The control group received no treatment, while the DGNTG groups were treated with two or 4 mg/mL DGNTG. Photographs were captured at 0, 12, and 24 h post-scratch using an inverted microscope (Olympus Corporation, Japan). The area of each wound was quantified for all images utilizing the wound healing size tool in ImageJ. The migration rate at 12 and 24 h was calculated using the formula: (0 h scratch area - 12 h/24 h scratch area)/0 h scratch area. Each experiment was conducted in triplicate.

### 2.7 The transwell assay

The impact of DGNTG on the invasiveness of MH7A cells was evaluated using the transwell assay. Matrigel was diluted with DMEM at a ratio of 1:8 and subsequently coated onto the transwell inserts, which were incubated overnight. Following this, MH7A cells (1 × 10^5^ per well) cultured in DMEM without FBS were seeded into the upper chambers of the transwell and treated with or without DGNTG (2 and 4 mg/mL) for 24 h at 37 °C, while the lower chambers contained DMEM supplemented with 20% FBS. After the incubation period, invasive cells within the transwell were fixed with 75% ethanol at room temperature for 30 min. These cells were then washed with PBS and stained with 0.1% crystal violet for 2 h. Finally, images were captured using an inverted microscope, and three fields were randomly chosen for cell counting. The assay was repeated on three separate occasions.

### 2.8 Western blotting assay

The MH7A cells were cultured overnight in a 6-well plate (2 × 10^5^ cells per well). Subsequently, the cells were treated with or without DGNTG (2 and 4 mg/mL) for 24 h at 37 °C. Total protein was extracted from the cells using RIPA lysis buffer, and the protein concentration was quantified with a Bradford protein assay kit. A total of 30 µg from each sample was separated by SDS-PAGE on a 12% gel (80 V for 20 min followed by 200 V for an additional 45 min). The proteins were subsequently transferred to a polyvinylidene fluoride membrane (0.22 μm) at a current of 300 mA for 1.5 h. The membrane was blocked with a solution containing 5% skim milk for 4 hours at room temperature before being incubated overnight at 4 °C with primary antibodies against β-actin (Rabbit, AB_2923704, 81115-1-RR, Proteintech, 1:5000), MMP9 (Rabbit, R380831, Zenbio, 1:1000), and MMP2 (Mouse, AB_2881746, 66366-1-Ig, Proteintech, 1:3000). Following this, the membranes were exposed to secondary antibodies of anti-rabbit (Goat, AB_2839429, S0001, Affinity, 1:6000) or anti-mouse (Goat, AB_2839430, S0002, Affinity, 1:5000) at room temperature for 2 h. Finally, visualization was performed using ProteinSimple FluorChem E (ProteinSimple, USA). The resulting images were quantified using ImageJ software and normalized against β-actin levels.

### 2.9 CeRNA microarray analysis

The MH7A was cultured in 10 cm dishes (1 × 10^7^ cell/dish) and treated with or without DGNTG (4 mg/mL) for subsequent ceRNA microarray analysis. Sample labeling and array hybridization were performed according to the established microarray protocol. In brief, total RNA was extracted using Trizol, and RNA integrity was assessed by evaluating the RIN number with an Agilent Bioanalyzer 2100. The RNA was then amplified and transcribed into fluorescent complementary RNA (cRNA), which was subsequently purified with the RNeasy Mini Kit. Following the manufacturer’s instructions, each slide was hybridized with 1.65 μg of cRNA utilizing the Gene Expression Hybridization Kit in a Hybridization Oven set to 65 °C, rotating at 10 rpm for 17 h. After hybridization, the arrays were washed, fixed, and scanned. Raw data normalization was conducted using the Quantile algorithm within the limma packages in R software. Differentially expressed (DE) genes were screened based on fold change (FC) and Student’s t-test. The selection criteria included: Fold Change (linear) ≤ 0.5 or Fold Change (linear) ≥ 2, and *t-test P < 0.05*.

### 2.10 GO and KEGG enrichment analysis

Utilizing the R software package (Yu et al., 2012) along with associated packages, we conducted Gene Ontology (GO) and Kyoto Encyclopedia of Genes and Genomes (KEGG) pathway enrichment analyses on the differentially expressed host circRNAs and their target mRNAs. For data analysis and visualization, we utilized the online platform SRplot (Tang et al., 2023). The GO analysis identified terms related to biological processes (BP), cellular components (CC), and molecular functions (MF). These terms elucidate the potential molecular functions of gene products, emphasizing the biological processes involved and the cellular environments in which they operate. Consequently, GO analysis provides a comprehensive overview of the functional attributes of genes and their products within organisms. KEGG pathway enrichment classifies known genomic annotation information while revealing significantly enriched signaling pathways.

### 2.11 Validation by real-time qPCR

The RNA samples remaining after microarray analysis were validated through qRT-PCR. HiScript III RT SuperMix for qPCR was subsequently utilized to synthesize cDNA, which was then amplified according to the instructions provided with ChamQ SYBR qPCR Master Mix. Five differentially expressed mRNAs and five circRNAs identified by microarray analysis were randomly selected, with GAPDH serving as the reference gene. Relative gene expression levels were calculated using the 2^−ΔΔCT^ method. The primer sequences are detailed in Table 2.

**TABLE 2:** The primer sequences used for real-time quantitative PCR.

Human gene	Forward primer	Reverse primer
hsa_circ_0065740	GCC​AGG​GCT​GTA​TGG​AAT​AA	CCG​GAT​ACA​CAC​ACC​ATC​TT
hsa_circ_0066830	TGC​AAG​TAT​GGC​CTG​TAC​GTC	CCA​TAA​ATG​TCT​CCG​CCA​GTG
hsa_circ_0077895	CGT​ACA​ATC​CTT​CCC​TCA​TCC​C	GTG​AAT​GCA​GGT​AGC​CCA​AG
hsa_circ_0067728	TCT​GGG​ATA​CAG​TCT​AAC​TCA​TCT​G	AGA​ACA​AAC​TGG​ACA​CAC​TCC​A
hsa_circ_0013495	TTG​CCC​CAA​ATC​TCA​TCA​CCA	AAT​ATC​GCC​TCC​GAC​TCC​AG
SGK1	AGG​ATG​GGT​CTG​AAC​GAC​TTT	GCC​CTT​TCC​GAT​CAC​TTT​CAA​G
CEPT1	CCC​CAA​ATC​TCA​TCA​CCA​TC	CTT​CTT​GCC​TGT​TTC​CCA​TC
RPL5	TCC​GCA​GGA​TGG​GGT​TTG​T	ACCAAGCGTTTCCGAGCA
FOXO6	CGA​CCT​CAT​CAC​CAA​AGC​CA	TGT​GCC​GGA​TGG​AGT​TCT​TC
ZANFD5	CAC​TCA​GCC​CAG​TCC​ATC​AG	TCG​GCA​GTC​AAA​CCC​TGT​AA
GAPDH	GAACGGGAAGCTCACTGG	GCC​TGC​TTC​ACC​ACC​TTC​T

### 2.12 The prediction of miRNA

The intersection of the DE mRNA and the DE circRNA host gene was taken. Subsequently, we collected the circRNA corresponding to these genes and conducted miRNA prediction for them. The circinteractome platform (Dudekula et al., 2016) was utilized to predict microRNA, from which we selected those with the highest context and score as our forecasting results.

### 2.13 Construction of the circRNA-miRNA-mRNA network

Based on the ceRNA hypothesis, a circRNA-associated ceRNA network was established as follows. First, coexpression relationships among miRNA-circRNA, miRNA-mRNA, and circRNA-mRNA pairs were calculated. The regulatory interactions of these screened coexpression pairs were predicted to enhance result accuracy and improve prediction reliability. Subsequently, the circRNA-miRNA-mRNA interaction network was constructed by importing data into Cytoscape in accordance with to the interaction framework. Finally, a reliable ceRNA relationship pair was identified.

### 2.14 Statistical analysis

A minimum of three independent measurements were obtained, and all data are presented as the *mean ± standard error of the mean (SEM).* One-way ANOVA or Student’s *t-tests* were employed for statistical analysis, with *P < 0.05* indicating statistically significant differences. SPSS version 26.0 and GraphPad Prism version 9.0 software were utilized for data analysis and processing.

## 3 Results

### 3.1 Effects of DGNTG on proliferation in MH7A cells

**FIGURE 2:**
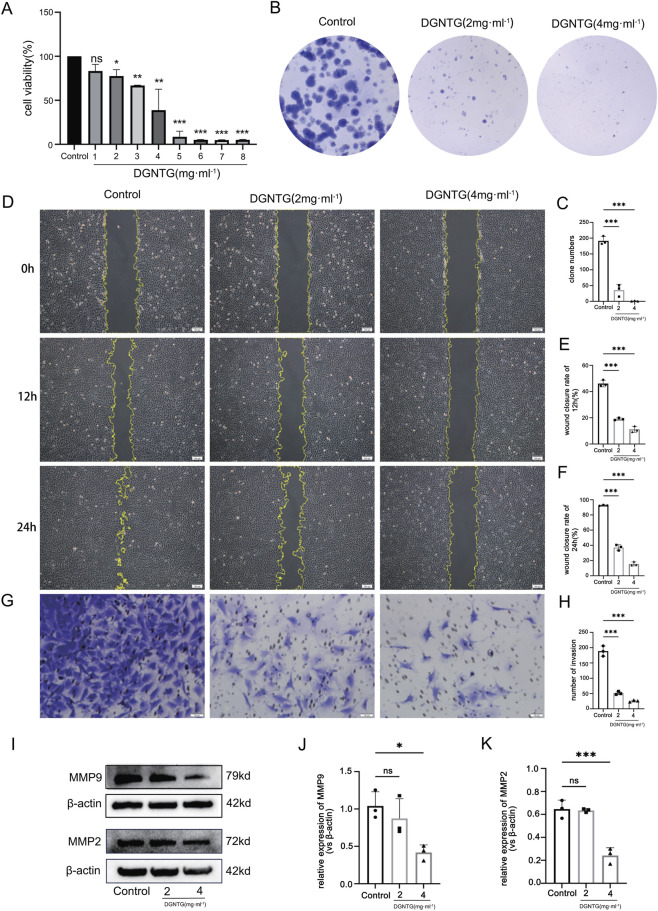
Effects of DGNTG on MH7A cells. **(A)** The MTT assay of MH7A cells incubated with DGNTG (0–8 mg/mL); **(B,C)** The represented pictures and statistical results of colony formation assay of MH7A cells treated by DGNTG (0, 2, 4 mg/mL); **(D–F)** The represented pictures and statistical results of wound healing assay of MH7A cells treated with DGNTG (0, 2, 4 mg/mL) for 12 and 24 h (×40); **(G,H)** The represented pictures and statistical results of transwell assay of MH7A cells treated with DGNTG (0, 2, 4 mg/mL) (×200); **(I–K)** Western blotting represented pictures and statistical results of MMP9 and MMP2 in DGNTG-treated MH7A cells; All data are presented as *mean ± SEM*, *n = 3* for each group. *P < 0.05, **P < 0.01, ***P < 0.001.

The MTT assay was performed to assess the impact of DGNTG on MH7A proliferation. MH7A were exposed to serial concentrations of DGNTG, ranging from 0 to 8 mg/mL for a duration of 24 h. The results demonstrated that DGNTG suppressed MH7A proliferation in a concentration-dependent manner (Figure 2A) (*P < 0.05, 0.01 or  0.001*), with the half-maximal inhibitory concentration (IC 50) was calculated to be 3.316 mg/mL. Consequently, concentrations of 2 and 4 mg/mL DGNTG were selected in the subsequent experiments. Furthermore, the colony formation assay, which reflects the proliferative capacity of cells, was employed. As illustrated in Figures 2B,C. The clone numbers significantly decreased in cells treated with DGNTG at concentrations of 2 and 4 mg/mL (*P <  0.001*).

### 3.2 Effects of DGNTG on migration and invasion in MH7A cells

The wound healing and transwell assays are established methods for assessing cell migration and invasion. The results demonstrated that MH7A cells in the control groups displayed significant migratory and invasive activity. In contrast, treatment with DGNTG (2 and 4 mg/mL) markedly inhibited both their migration and invasion (*P < 0.001*), as shown in Figures 2D–H.

### 3.3 Effect of DGNTG on expression of MMP9 and MMP2 in MH7A cells

Matrix metalloproteinases (MMPs) are known to degrade the extracellular matrix and alter intercellular adhesion (Asgari et al., 2023). Consequently, MMPs are pivotal in the pathological processes involving the migration and invasion of fibroblast-like synovial cells in RA. To evaluate the expression levels of MMP9 and MMP2, a Western blotting assay was conducted. As shown in Figures 2I–K, treatment with 2 mg/mL DGNTG resulted in only a modest decrease in the expression of MMP9 and MMP2(*P > 0.05*). And 4 mg/mL DGNTG obviously inhibits MMP9 and MMP2 expression (*P < 0.01 or  0.001*). Therefore, 4 mg/mL DGNTG was chosen as the concentration for subsequent experiments.

### 3.4 Analysis of differentially expressed mRNAs

**FIGURE 3:**
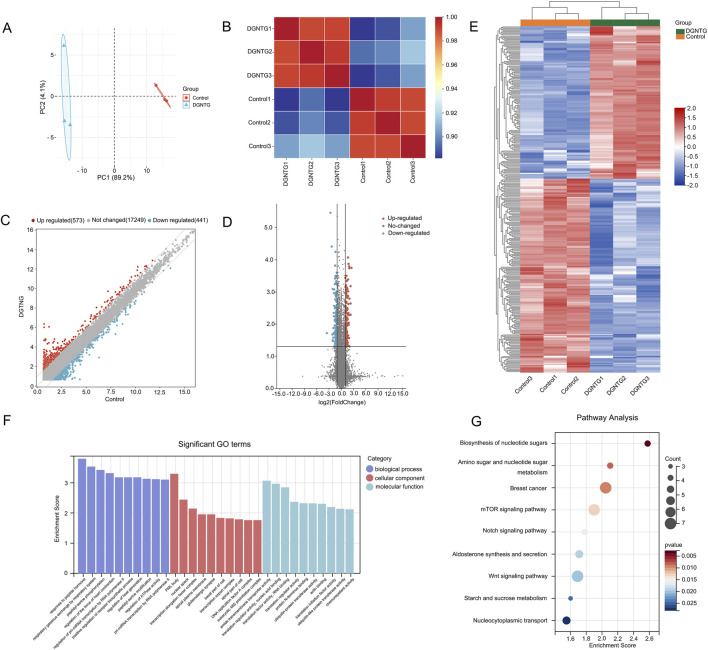
DE mRNAs and functional enrichment analysis. **(A)** Principal component analysis of mRNAs; **(B)** The correlation analysis of mRNAs; **(C)** The scatter plot of the expression profiles of DE mRNAs; **(D)** The volcano plot of the expression profiles of DE mRNAs; **(E)** Heat map about DE mRNAs; **(F)** GO enrichment analysis of DE mRNAs; **(G)** KEGG enrichment analysis of DE mRNAs.

RNA was extracted from three control samples and three DGNTG-treated samples, followed by an analysis of mRNA expression profiles. Principal component analysis (PCA) demonstrated a significant difference between the DGNTG group and the control group (Figure 3A). The correlation heatmap revealed a high degree of correlation among samples at the mRNA level (Figure 3B). A total of 18,263 probes were utilized to assess mRNA levels. In comparison to the control group, the DGNTG group exhibited 133 upregulated and 168 downregulated mRNAs (Figures 3C–E). GO function and KEGG pathway enrichment analyses were performed for the mRNAs targeted by DGNTG. As shown in Figure 3F, these targeted mRNAs were primarily enriched in transcriptional regulation processes, including pri-miRNA transcription by RNA polymerase II of BP terms, and transcription elongation factor complex of CC terms. The KEGG pathway enrichment analysis indicated that Wnt signaling pathway, mTOR signaling pathway, Notch signaling pathway, and Biosynthesis of nucleotide sugars may be closely associated with the effect of DGNTG in MH7A (Figure 3G).

### 3.5 Analysis of differentially expressed circRNAs

**FIGURE 4:**
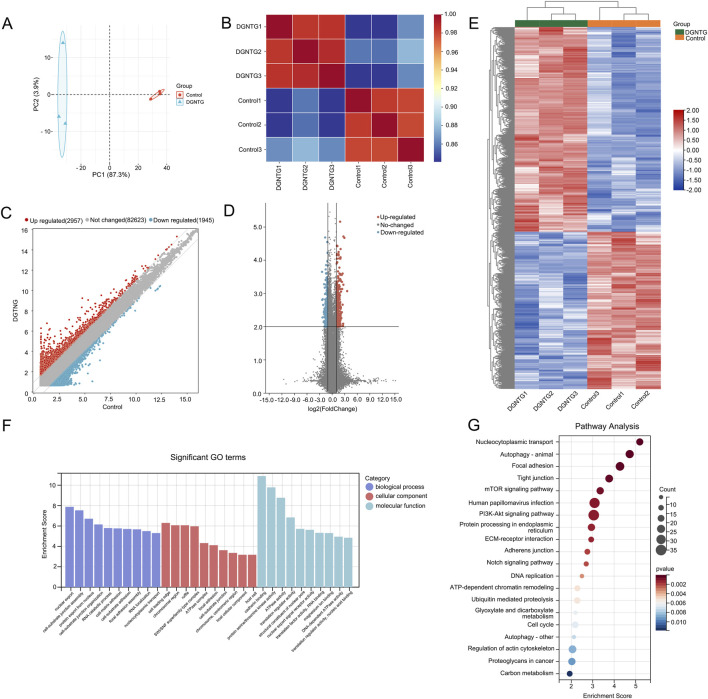
DE circRNAs and functional enrichment analysis. **(A)** Principal component analysis of circRNAs; **(B)** The correlation analysis of circRNAs; **(C)** The scatter plot of the expression profiles of DE circRNAs; **(D)** The volcano plot of the expression profiles of DE circRNAs; **(E)** Heat map about DE circRNAs; **(F)** GO enrichment analysis of host genes of DE circRNAs; **(G)** KEGG enrichment analysis of host genes of DE circRNAs.

We subsequently examined the alteration in circRNA expression. A total of 87,525 probes were used to generate circRNA profiles. The PCA results demonstrated a distinct difference in circRNA expression between the DGNTG group and the control group (Figure 4A). Additionally, a relatively high correlation was noted between these two groups (Figure 4B). Following DGNTG intervention, we identified a total of 1,507 differentially expressed circRNAs, of which 854 were upregulated and 653 were downregulated (Figures 4C–E). We then conducted enrichment analysis on the host genes associated with these differentially expressed circRNAs. The enriched GO analysis primarily pertained to cell matrix and adhesion processes, including cell-matrix adhesion and cell-substrate adhesion in BP, as well as focal adhesion and cell-substrate junction in CC terms (Figure 4F). The result of KEGG pathway enrichment analysis primarily encompassed the Autophagy-animal, RNA transport, mTOR signaling pathway, Notch signaling pathway, and PI3K/Akt signaling pathway, as depicted in Figure 4G.

### 3.6 Construction of a ceRNA network

**FIGURE 5:**
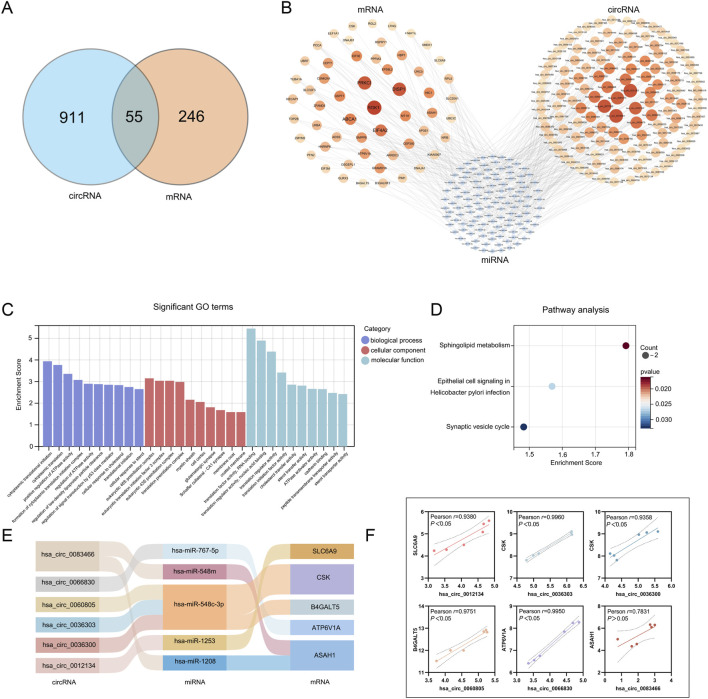
Establishment of the ceRNA network. **(A)** The intersection of DE mRNAs and the host genes of DE circRNAs; **(B)** The interaction network of circRNA-miRNA-mRNA: the colors of mRNAs and circRNAs are shaded from light to dark according to their foldchange (abs). **(C)** GO enrichment analysis of 55 genes; **(D)** KEGG enrichment analysis of 55 genes; **(E)** Sankey diagram for the representative ceRNA network; **(F)** Pearson correlation analysis between mRNAs and circRNAs.

First, a total of 957 host genes of differentially expressed circRNAs were found. 55 genes were obtained by the intersection of the results from DE mRNAs and the host genes of DE circRNAs (Figure 5A) and 126 circRNAs corresponded to them. Based on circinteractome database a total of 116 miRNAs were predicted.

Based on the DE RNA profiles identified between the DGNTG and control groups, a circRNA-miRNA-mRNA network associated with DGNTG was constructed (Figure 5B). This network comprised 297 nodes (containing 126 circRNAs, 116 miRNAs, and 55 mRNAs) and 343 edges. Additionally, the GO and KEGG analyses were performed. As shown in Figure 5C, these genes were linked to cell energy metabolism and cytoskeletal composition, specifically highlighting the positive regulation of ATPase activity in BF terms and cell cortex in CC. Interestingly, KEGG analysis revealed only three enriched pathways (Figure 5D). Subsequently, we identified the mRNAs along with their circRNAs and miRNAs that enriched these pathways to construct a representative ceRNA network (Figure 5E). This representative ceRNA network consisted of six circRNAs (hsa_circ_0083466, hsa_circ_0036303, hsa_circ_0036300, hsa_circ_0012134, hsa_circ_0060805, hsa_circ_0066830), five miRNAs (hsa-miR-1208, hsa-miR-548m, hsa-miR-548c-3p, hsa-miR-1253, hsa-miR-767-5p) and five mRNAs. Pearson correlation analysis was employed to evaluate the expression relationships between circRNAs and their corresponding host mRNAs (Figure 5F). The results indicated that five circRNAs exhibited a significant positive correlation with their corresponding host mRNAs(*P < 0.05*). Although no statistical difference between hsa_circ_0083466 and ASAH1, the Pearson correlation coefficient suggested a moderate correlation.

### 3.7 Validation of the microarray data

**FIGURE 6:**
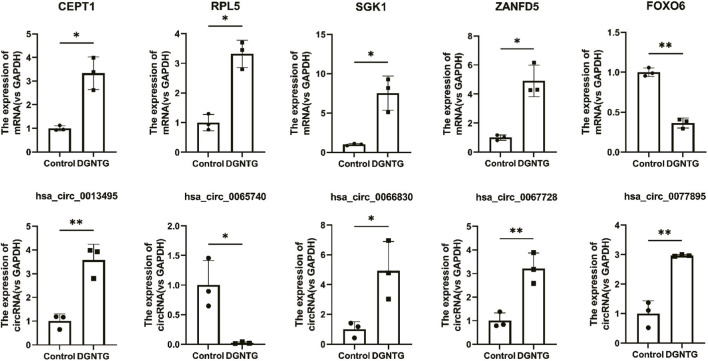
Validation of the microarray data by RT-qPCR. All data are presented as *mean ± SEM*, *n = 3* for each group. *P < 0.05, **P < 0.01.

Five mRNAs (CEPT1, RPL5, SGK1, ZANFD5, FOXO6) and five circRNAs (hsa_circ_0013495, hsa_circ_0065740, hsa_circ_0066830, hsa_circ_0067728, hsa_circ_0077895) were randomly selected for RT-qPCR analysis to validate the expression changes identified through microarray analysis. As illustrated in Figure 6, the expression levels determined by RT-qPCR were consistent with the trends observed in the microarray data (*P < 0.05 or  0.01*), thereby confirming the accuracy of the microarray results.

## 4 Discussion

RA is a prevalent clinical disease characterized by a complex pathogenesis and the absence of a specific treatment. The prolonged treatment duration and severe sequelae impose a substantial burden on both patients and society. As a complementary therapy, TCM is gaining traction in the management of RA; however, the theoretical molecular mechanisms underlying its efficacy remain inadequately supported. Our study demonstrates that DGNTG significantly inhibits the proliferation, migration and invasion of fibroblast-like synoviocytes MH7A. These beneficial effects may be mediated through the circRNA-miRNA-mRNA transcriptional network.

*In vivo* experiments and clinical trials have confirmed the therapeutic efficacy of DGNTG for RA; however, the underlying cellular and molecular mechanisms remain unclear. Abnormal proliferative synovial fibroblast-like cells are among the primary pathological cell types involved. These cells secrete inflammatory transmitters (IL-6, IL-1β, TNF-α), which initiate an inflammatory cascade, invade cartilage and exacerbate RA symptoms (Masoumi et al., 2021). Inhibiting synovial hyperplasia and migration constitutes an effective strategy for treating rheumatoid arthritis. The MH7A cell line serves as a well-established disease-specific model for rheumatoid arthritis fibroblast-like synoviocytes, which are characterized by aberrant, tumor-like proliferation and invasion (Lin et al., 2024). In this study, we employed MH7A to investigate the efficacy of DGNTG. Our results demonstrated that DGNTG significantly inhibits cell proliferation while suppressing migration and invasion in MH7A cells, thereby underscoring its beneficial effects on RA. Furthermore, MMPs, which are key proteases involved in the invasion and degradation of basement membranes and extracellular matrices, are closely associated with cellular migration and invasion capabilities (Sleeboom et al., 2024). Among the MMP family members, MMP9 and MMP2, produced by FLS play crucial roles in degrading fibrillar collagens and extracellular matrix components in RA (Bian et al., 2023). Therefore, we posit that DGNTG’s inhibitory effect on MMP9 and MMP2 expression is advantageous for its suppression of MH7A cell migration and invasion. In our study, we observed that DGNTG significantly suppressed MMP9 and MMP2 expression. Based on these findings, we speculate that DGNTG inhibits the migration and invasion of FLS, thereby alleviating symptoms associated with RA progression. However, the precise molecular mechanisms by which DGNTG exerts its effect in RA remain to be fully elucidated.

High-throughput detection methods utilizing gene chips can be employed to investigate the therapeutic mechanisms of drugs at the whole transcriptome level. These methods have increasingly been applied to identify specific markers of traditional Chinese medicine syndromes and to study the therapeutic mechanisms of TCMs (Chen et al., 2022). In this study, we identified 133 upregulated and 168 downregulated mRNAs in DGNTG-treated MH7A cells. These targeted mRNAs were primarily associated with transcriptional regulation, suggesting their involvement in the regulation of gene expression influenced by DGNTG in MH7A. Further analysis revealed the potential implication of the Wnt signaling pathway, mTOR signaling pathway, and Notch signaling pathway in mediating the effects of DGNTG. The Wnt signaling pathway contributes to cellular proliferation, inflammation, migration, and invasion in rheumatoid arthritis fibroblast-like synoviocytes, making its suppression a promising therapeutic target for RA (Cai L et al., 2022; Liu et al., 2023). The mTOR signaling pathway is linked to RA through immune and metabolic signals, including immune cell proliferation and differentiation (Zhang et al., 2023). Additionally, Notch signaling plays a crucial role in hypoxia-induced angiogenesis and activates FLS in rheumatoid arthritis (Chen et al., 2021; Zhao et al., 2023).

As a CeRNA, circRNA acts as a miRNA sponge, thereby modulating the expression of mRNAs. In this study, we identified a total of 1,507 DE circRNAs following DGNTG intervention, of which 854 being upregulated and 653 downregulated. The GO enrichment revealed that these DE circRNAs were primarily associated with cell matrix and adhesion processes, which are correlated with cell migration and invasion. Collectively, these findings suggest that DGNTG can modulate the proliferation, migration, and invasion of MH7A cells. Notably, the DE circRNAs were enriched in pathways such as mTOR signaling and Notch signaling as indicated by mRNA analyses. Moreover, circRNAs were found to be enriched in Autophagy - animal and PI3K-Akt signaling pathway, which may be closely related to the anti-RA mechanism of DGNTG. In the pathogenesis of RA, autophagy is implicated in the maturation, survival, and proliferation of various immune and non-immune cells (Karami et al., 2020). It has been reported that a reduction in autophagy alleviates inflammation in rheumatoid arthritis (Xiao et al., 2024). Actually, we have previously demonstrated that DGNTG can reduce synovial inflammation by inhibiting autophagy (Lu et al., 2022a). The PI3K/Akt signaling pathway plays an essential role in the pathogenesis of numerous diseases. It is a classical pathway regulating cellular metabolism and apoptosis. In the context of RA, abnormal activation of this pathway can lead to excessive proliferation of FLS (Cheng et al., 2023; He et al., 2023). Our prior studies have shown that DGNTG increases FLS apoptosis through PI3K/Akt pathway *in vivo* (Cai Y et al., 2022). In conclusion, the aforementioned biological processes and signaling pathways may represent therapeutic targets of DGNTG in RA.

Furthermore, we identified 55 genes by intersecting the results from DE mRNAs and the host genes of DE circRNAs. Analysis of these 55 genes revealed that many of them influence cell proliferation, migration, and invasion, including HBP1, MT1H, RGL2, SLC6A9, LFNG, B4GALT5, and GMPPB. Studies have demonstrated that HBP1 can inhibit the growth and inflammation of rheumatoid arthritis synovial fibroblasts (Wang et al., 2021) while MT1H suppresses the proliferation, invasion, and migration of hepatocellular carcinoma cells by regulating the Wnt/β-catenin signaling pathway (Zheng et al., 2017). In this study, the results indicated that DGNTG upregulates the expression of both HBP1 and MT1H. According to the reports, RGL2, SLC6A9, LFNG, B4GALT5 and GMPPB are positively correlated with cellular proliferation, migration and invasion. Knockdown of RGL2 markedly impairs the metastatic potential of colorectal cancer cells both *in vivo* and *in vitro* (Sun et al., 2021). Additionally, the suppression of glycine utilization via the SLC6A9 knockdown inhibits the proliferation in multiple myeloma cells (Xia et al., 2022). Overexpression of LFNG enhances cell proliferation and invasion (Gong et al., 2023). Conversely, deletion of B4GALT5 significantly reduces proliferation, migration, and invasion in hepatocellular carcinoma cells (Han et al., 2022), while silencing GMPPB diminishes glioblastoma cell proliferation, migration, and invasion both *in vitro* and *in vivo* (Huang et al., 2023). In the present study, downregulation of RGL2, SLC6A9 and LFNG, B4GALT5, and GMPPB were observed after treatment with DGNTG. Therefore, we hypothesize that DGNTG may inhibit proliferation, migration and invasion of synovial fibroblast by modulating the expression levels of these genes. Additionally, several genes are closely related to apoptosis and autophagy. These processes also impact synovial fibroblast behavior in terms of proliferation, migration and invasion. For instance, our results indicated increased expression levels of CSNK2A1 and SGK1 after DGNTG treatment. Previous reports suggest that CSNK2 can suppress autophagy through activation of FLN-NHL-containing TRIM proteins (Hoenigsperger et al., 2024), while SGK1 is known to enhance reactive oxygen species (ROS) production, leading to apoptosis (Liu et al., 2024). Thus, DGNTG may reduce autophagy while promoting apoptosis by upregulating the expression of CSNK2A1 and SGK1. Collectively, the above findings suggest that these genes can serve as potential therapeutic targets of DGNTG for RA.

MicroRNA (miRNA) is an evolutionarily conserved class of non-coding RNAs (ncRNAs) that play a pivotal role in gene regulation (He et al., 2020). Dysregulation of miRNAs is implicated in various human diseases due to abnormalities in biological processes. In this study, we predicted 116 miRNAs corresponding to the 55 genes mentioned above. These miRNAs are associated with cell proliferation, migration, and invasion. For instance, hsa-miR-767-5p functions as an oncogenic driver in non-small cell lung cancer by targeting MAPK4, thereby facilitating tumor growth (Yang et al., 2021). In hepatocellular carcinoma, miR-548m is sequestered by the circular RNA circBPTF, leading to the upregulation of POLR3G and ZBTB41 proteins, which ultimately promote cancer cell invasion and migration (Yang et al., 2025). Conversely, miR-548c-3p is upregulated in castration-resistant prostate cancer, thereby enhancing proliferative and invasive potential (Feng et al., 2019). In contrast, miR-1253 serves as a tumor suppressor in osteosarcoma, where its overexpression inhibits proliferation, migration, and invasion through direct targeting of MMP9, a key enzyme involved in extracellular matrix degradation (Mo et al., 2021). Similarly, miR-1208 is suppressed by the lncRNA TMEM105, resulting in LDHA upregulation and enhanced glycolysis, which fuels cancer cell invasion and liver metastasis; thus, miR-1208 acts as a progression inhibitor (Han et al., 2023). These findings collectively highlight the dual roles of miRNAs as critical regulators of biological processes such as proliferation, migration, and invasion, consistent with our study.

In addition, We ultimately integrated the DE circRNAs, DE mRNAs and miRNAs into a circRNA-miRNA-mRNA ternary transcription network, which comprises 126 circRNAs, 116 miRNAs, and 55 mRNAs. The GO enrichments indicated that DGNTG may exert its therapeutic effects on MH7A by regulating cellular energy metabolism and cytoskeletal composition. Notably, there were only three pathways enriched by KEGG analysis. To further investigate the relationship within the ceRNA network, we identified the mRNAs alongside their corresponding circRNAs and miRNAs that enriched these pathways, thereby constructing a representative ceRNA network. This network exhibits three distinct characteristics: a circRNA associated with multiple miRNAs, a miRNA binding to various mRNAs, and an mRNA corresponding to different circRNAs. Additionally, we performed Pearson correlation analysis on these six circRNAs and five mRNAs. The results demonstrated that six circRNAs were positively correlated with their host mRNAs, thereby confirming both the accuracy and high correlation of our ceRNA network.

This study employed an integrated transcriptomic approach to investigate the effects of DGNTG on the rheumatoid arthritis fibroblast-like synoviocyte cell line MH7A, focusing primarily on the construction of a circRNA-miRNA-mRNA regulatory network. A principal strength of this work lies in the simultaneous analysis of mRNAs, circRNAs, and miRNAs, which has enabled the proposal of a potential ceRNA network associated with DGNTG treatment. This network provides a testable hypothesis for elucidating RNA interactions at the transcriptional level that may underlie the pharmacological effects of DGNTG. Consequently, our findings provided a preliminary framework for elucidating the molecular mechanisms of DGNTG in RA, thereby establishing a foundation for future functional validation studies and potential clinical exploration. However, it is important to acknowledge the limitations of this study. Our analysis, while comprehensive, remains primarily predictive and did not experimentally pinpoint or functionally validate a core ceRNA axis. Therefore, future research will be essential to identify and verify the central regulatory axis within this network through rigorous *in vitro* and *in vivo* experiments.

## 5 Conclusion

In conclusion, this study provides evidence that DGNTG exhibits anti-RA effects associated with its inhibition of synoviocyte proliferation, migration and invasion. To explore the underlying mechanism, we constructed a ceRNA network based on transcriptomic data from DGNTG-treated MH7A cells. While this network offers a valuable resource for understanding DGNTG’s mechanism of action, future studies are required to functionally validate the core ceRNA axis and establish a direct causal link to the phenotypic outcomes.

## Data Availability

The data presented in the study are deposited in the NCBI (GEO) repository, accession number GSE318151.
